# Emergency Medicine Residents’ Perceptions of Working and Training in a Pandemic Epicenter: A Qualitative Analysis

**DOI:** 10.5811/westjem.2022.9.57298

**Published:** 2022-12-30

**Authors:** Adrian Aurrecoechea, Nidhi Kadakia, Jay V. Pandya, Marie J. Murphy, Teresa Y. Smith

**Affiliations:** SUNY Downstate Health Sciences University, Department of Emergency Medicine, Brooklyn, New York. NYC H+H Kings County Hospital, Department of Emergency Medicine, Brooklyn, New York

## Abstract

**Introduction:**

We sought to describe the range of emergency medicine (EM) resident physicians’ perceptions and experiences of working and training during the initial coronavirus 2019 (COVID-19) pandemic surge at two, large-volume, urban training hospitals in Brooklyn, New York.

**Methods:**

A total of 25 EM resident physicians who worked at either of two large emergency departments (ED) from March 15–April 11, 2020 participated in semi-structured interviews conducted in July and August 2020. Interviews were conducted by the authors who were also emergency medicine resident physicians working in the ED during this time. We asked open-ended questions to residents about their experiences and emotions at work and outside of work, including their relationship with co-workers, patients, and their community. The interviews were audio-recorded and transcribed. We then conducted a thematic analysis to identify, classify, and define themes from interview transcripts. Iterative commonalities and differences between interview response themes were grouped to create a broadly applicable narrative of the residents’ perceptions and experiences of working and training during this initial wave of a novel pandemic. Interviewees also responded to a demographics survey.

**Results:**

Study participants described four major aspects of their perceptions and experiences of working and training during the stated time, including emotional challenges such as anxiety and feeling underappreciated; protective thoughts, including camaraderie, and sense of duty; workplace challenges such as limited knowledge surrounding COVID-19 and a higher volume of acute patients; and adaptive strategies including increased communication with ED administrators.

**Conclusion:**

Emergency medicine residents have a unique perspective and were key frontline hospital responders during a prolonged disaster and mass triage event within a local health system. Considering the chronic case and mortality fluctuations and new variants of COVID-19, as well as the anticipation of future infectious disease pandemics, we believe it is important for key decision-makers in resident education, hospital administration, and all levels of public health management to inform themselves about residents’ emotional and workplace challenges when establishing hospital and residency program disaster protocols.

## INTRODUCTION

On March 11, 2020, the same day that the World Health Organization declared coronavirus 2019 (COVID-19) a pandemic, New York City (NYC) had its first confirmed COVID-19 associated fatality.^1^,^2^ According to the US Centers for Disease Control and Prevention, from February 29–June 1, 2020 there were 203,792 confirmed diagnoses of COVID-19 in NYC. Of those patients with confirmed diagnoses, 54,211 were hospitalized and 18,679 died.^3^ Hospital admissions peaked in NYC the week of March 29, with a mean of 1,566 admissions/day. Deaths peaked in NYC the week of April 5 with 566 deaths/day.^3^ The increase in hospital admissions and emergency department (ED) visits placed increased stress on an already strained healthcare system and clinicians.

Prior qualitative research has reported on the healthcare worker experience during pandemics and natural disasters. These studies have primarily focused on the psychological wellbeing of staff exposed, the healthcare worker experience, and the attitudes and willingness of healthcare workers toward coming to work.^4^–^8^ In 2016, a systematic review was published looking at 111 papers to understand steps that can be taken at all stages of a disaster (before, during, and after), which may minimize risks to responders and enhance resilience including preparedness and support.^9^ However, there has been limited research published focusing specifically on postgraduate trainees’ perceptions of their experience working, learning, and living through any pandemic, including COVID-19. In fact, although thousands of papers have been published between 2020–2021 regarding COVID-19, only a handful were qualitative studies.

There has yet to be published participant observation research exploring the details of resident experience early in the COVID-19 pandemic with significant depth. This is likely due to time constraints; however, in doing so, this has “[hindered] the exploration and portrayal of complex human and social phenomena and therefore [produced] less credible findings due to short-term immersion between the researcher and participants.”^10^ In this paper we explore emergency medicine (EM) residents’ perceptions of working and training during the first wave of the COVID-19 pandemic in two urban hospitals using participant observation, where investigators had been completely integrated into the study population beforehand. The information from this study can be used by EM residents, residency directors, hospital administrators, and emergency preparedness professionals to help hospitals, residency programs, and residents/trainees globally prepare for future pandemics, and natural and/or manmade disasters.

## METHODS

### Study Design

The authors (excluding TS, the principal investigator [PI]), were EM resident physicians working in the ED during the dates of interest and at the time interviews were conducted. We interviewed EM residents who worked primarily at one public urban, safety-net, large-volume Level I trauma center and/or at a separate tertiary care center designated as a COVID-19 only facility. The PI was an attending emergency physician at the study sites during the dates of interest and at the time interviews were conducted. These hospitals saw an influx of patients during a time when New York City was described as a COVID-19 epicenter during the first wave of the COVID-19 pandemic in the US.

**Population Health Research Capsule**

What do we already know about this issue?

*Recent qualitative research describes negative emotions and interpersonal relationships of emergency medicine (EM) residents working outside the US during the coronavirus disease 2019 (COVID-19) pandemic*.

What was the research question?

*What were EM residents’ perceptions and experiences of working and training during the initial COVID-19 pandemic?*

What was the major finding of the study?

*EM residents have a unique perspective during prolonged disaster and mass triage events within a local health system*.

How does this improve population health?

*The results from our study will help hospitals, residency programs, and residents/trainees globally prepare for future pandemics, and natural and/or manmade disasters*.

We conducted a thematic analysis of interview data using qualitative methodology to bring out rich and meaningful narratives of this group’s experiences during dates of high utilization of emergency medical services.^11^ We selected and finalized the study design, including utilization of interviews, the interview content, timing, and analysis, for the purpose of identifying a range of themes de novo, centered around EM resident perceptions and experience of working and training during the initial COVID-19 pandemic surge at two, large-volume, urban training hospitals in Brooklyn, NY. Our academic institution’s institutional review board (IRB) approved the study. No conflicts of interest were identified in the IRB approval process.

### Participants

A total of 25 EM residents took part in one-on-one, semi-structured interviews conducted in July and August 2020. Two of the primary authors, junior residents in the residency under focus, recruited participants by emailing all residents who met inclusion criteria, informing them about the nature and purpose of the study. The recruitment information requested that all residents not interested in participating opt out and that participation was voluntary. The primary investigators involved in the study’s development were excluded from the study. Those interested in participating enrolled to participate in an interview. Of 97 EM residents in the 2019–2020 academic year, 73 met inclusion criteria, having worked at either urban hospital’s ED site between March 15, 2020–April 11, 2020 for at least one shift. The study’s authors were excluded from selection. Residents who met inclusion criteria were sorted by postgraduate year (PGY) class for the 2019–2020 academic year and into EM and EM/Internal Medicine (IM) combined residency (EM/IM) and subsequently randomized. We refer to these participants (both EM and EM/IM residents) as “emergency medicine residents” in this manuscript.

### Sampling

We recruited a purposive randomized representative sample of residents to participate, excluding those who did not work in the primary EDs, mentioned prior, during these dates or were involved in the study’s design. Of the 73 residents who met inclusion criteria, 25 were asked to participate. These 25 were selected by an online randomization tool that identified four random participants from each EM class, and one random participant from each EM/IM class. One PGY-1 EM resident declined to participate after randomization, and another resident who met inclusion criteria was randomly selected for that group. There was a total of 25 participants, broken down into five participants for each EM PGY class 1–4 and one participant for each EM/IM PGY class 1–5. We reviewed the list of interviewees prior to interviews by the investigators prior to interviews, and the sample was judged to be a sufficiently diverse (age, gender, ethnicity, hospital site, duration of time worked, and educational background) representation of the residency classes involved in the study.

### Survey and Interviews

Study participants responded to a demographics survey emailed to participants and administered via Google Forms. Individual interviews took place and confidentiality was ensured before the interview began. Additionally, the participant gave verbal consent before beginning the interview. Interviews were conducted by the four EM resident physician authors and excluding the PI/last author. The investigators had prior academic experience and training conducting qualitative interviews. Interview transcripts were reviewed weekly to assure quality and consistency of experience and data acquisition and to identify and correct any deviation. Interviews were 30–70 minutes long. The broad range of interview duration can be attributable to depth or brevity of participant responses despite two to four probing questions per core question. Interviews took place using online video platforms, Zoom and Google Meet. All interviews were audio-recorded and transcribed verbatim using the automated audio transcription software, Descript, (San Francisco, CA) and then corrected manually by the investigators.

All interviews followed the structure set out in the interview guide (Supplemental Materials), with minor iterative changes as interviews progressed. Generally, interviews began with “how are you feeling?” or “how has work been” as general ice-breaker questions. The subsequent question asked the subject to discuss their experiences working in the ED prior to COVID-19 peak dates, followed by an open-ended question: Describe working in the ED during the last two weeks of March and the first two weeks of April 2020. Subsequent questions asked the subject to describe their relationship with patients, ED administrators, ED attending physicians, other ED staff, and co-residents during the focus dates.

For the final 10 interviews, there was, at this point in the interview, a question about what was on a subject’s mind while arriving to shift and/or a question about home life during these focus dates. Residents were asked to describe the actions, if any, that were initiated in the workplace and the residency program for the purpose of quality improvement or wellness during the focus dates. Residents then were given the opportunity to add or clarify anything, and the interview concluded with a question about what advice the interviewee subjects would give to other residency program leadership and/or EM residents who found themselves in similar situations in the future. For each of these questions, the interviewers probed the interviewee if a response needed more clarification, elaboration, or examples.

### Data Analysis

Demographic survey data was analyzed via Microsoft Excel (Microsoft Corporation, Redmond, WA). We analyzed interviews using the qualitative analysis software MaxQDA (Verbi, Berlin, Germany), tallied conceptual themes mentioned by our participants, and consolidated and deduced from participant responses our general and evolving questions surrounding their experience in the ED during the first wave of COVID-19 in the US. Themes were derived from participant responses to emotion-neutral and opinion-neutral questions about workplace and out-of-workplace relationships and EM medicine trainee experiences during a prolonged disaster and mass triage event lasting approximately four weeks.

Themes found in the transcripts of the interviews were initially subdivided into the following primary categories: workplace challenges; adaptive workplace strategies; emotional challenges; and positive thoughts and resilience (later renamed protective thoughts), based on a consensus by the investigators to categorize what participants chose to discuss in their interview responses. The primary categories of relationships, clinical learning, and wellness activities were initially created inductively, as participants were asked to speak on these topics with open-ended, emotionally neutral questions. These categories were further divided into their final sub-thematic codes based on how participants chose to focus their answers to these questions.

When creating themes based on the spoken details of our interviews, we combined conceptually equivalent themes. This was true of sub-themes within all primary categories. For example, when transcript segments described the challenging emotions of “feeling unprepared,” “feeling overwhelmed,” or “feeling powerless” to describe the patient care experience, we initially coded these separately but ultimately combined these themes and recoded the transcript segments under the new theme: “Unprepared, overwhelmed, and/or powerless.” We maintained themes that were mentioned by greater than 10% of our informants. After an iterative review of theme names, thematic code definitions, and document transcripts, interrater reliability was good (Cohen’s kappa [k] = 0.82.). While the primary categories of relationships, clinical learning, and wellness activities were initially created, authors found that the relationships were not brought up as a primary theme of discussion by participants but rather brought up as subcategories of examples in the context of the major themes presented.

## RESULTS

### Study Participant Demographics

We interviewed 25 residents with multiple dimensions of demographic diversity (see Table 1). There was a total of 25 participants, broken down into five participants for each EM PGY class 1–4 and one participant for each EM/IM PGY class 1–5. Of those, 13 identified as women and 12 identified as men.

### Emotional Challenges and Protective Thoughts

**Figure 1:**
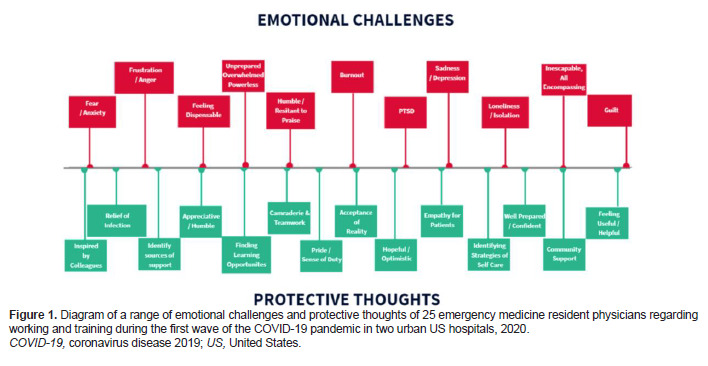
Diagram of a range of emotional challenges and protective thoughts of 25 emergency medicine resident physicians regarding working and training during the first wave of the COVID-19 pandemic in two urban US hospitals, 2020. *COVID-19*, coronavirus disease 2019; *US*, United States.

**Figure 2:**
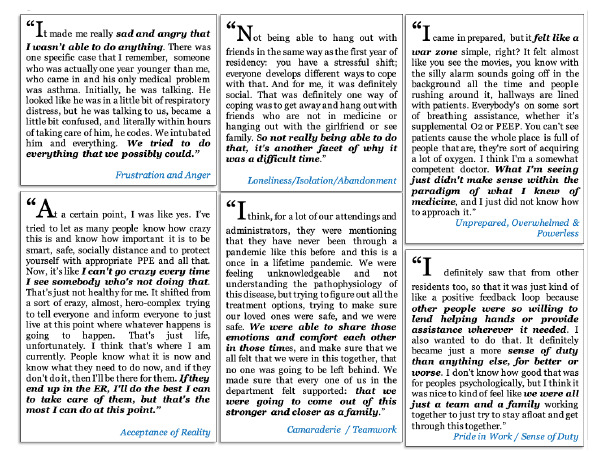
Quotations describing the emotional challenges and protective thoughts of 25 emergency medicine resident physicians regarding working and training during the first wave of the COVID-19 pandemic in two urban US hospitals, 2020. *COVID-19*, coronavirus disease; *US*, United States.

Emergency medicine residents recalled experiencing a wide range of challenging and protective emotions and thoughts related to their work environment. (See Figure 1 for a diagram of these themes and Figure 2 for a sample of quotes in these categories; a complete codebook of themes, definitions, and representative quotes can be found in the Supplemental Materials.) Challenging emotions included the following: feeling stress, fear and anxiety; frustration and anger; feeling underappreciated or dispensable; feeling unprepared, overwhelmed, or powerless; feeling trapped or unable to escape COVID-19; feeling humble or resistant to praise; feeling lonely, isolated, abandoned, misunderstood or excluded; feeling sad or depressed, feeling remorseful or guilty of personal decisions; feeling burned out, morally distressed, exhausted, apathetic, or numb; and feeling post-traumatic stress or secondary trauma.

Positive, protective, or resilient emotions and thoughts included the following: feeling inspired or proud of colleagues; feeling relieved by getting sick or wishing to get sick to avoid worrying about it; feeling appreciative or surprised in a positive way by certain outcomes; being able to find learning opportunities; feeling a sense of camaraderie or teamwork; feeling proud to work or a sense of duty; accepting of reality; feeling hopeful or optimistic; feeling empathetic toward patients or their families; identifying strategies for self-care; identifying sources of emotional support; feeling well-prepared, confident, or trusting of one’s self; feeling supported by the community; and finding ways to feel useful or helpful by being flexible with roles in the workplace or coordinating wellness activities and response efforts.

### Workplace Challenges and Adaptive Solutions

**Figure 3:**
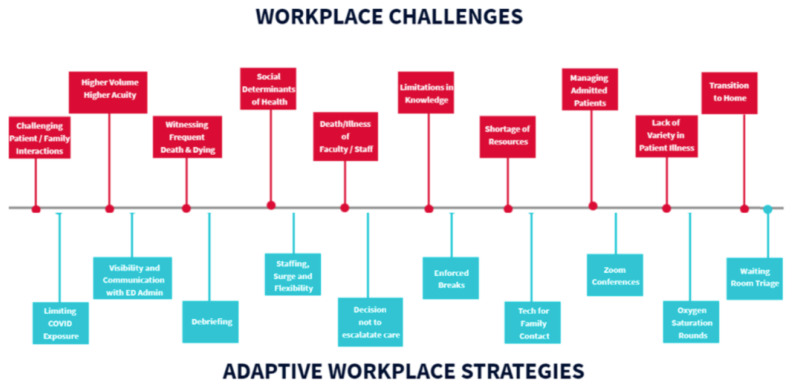
Diagram of a range of workplace challenges and adaptive workplace strategies experienced by 25 emergency medicine resident physicians when working and training during the first wave of the COVID-19 pandemic in two urban US hospitals, 2020. *COVID-19*, coronavirus disease 2019; *US*, United States.

**Figure 4:**
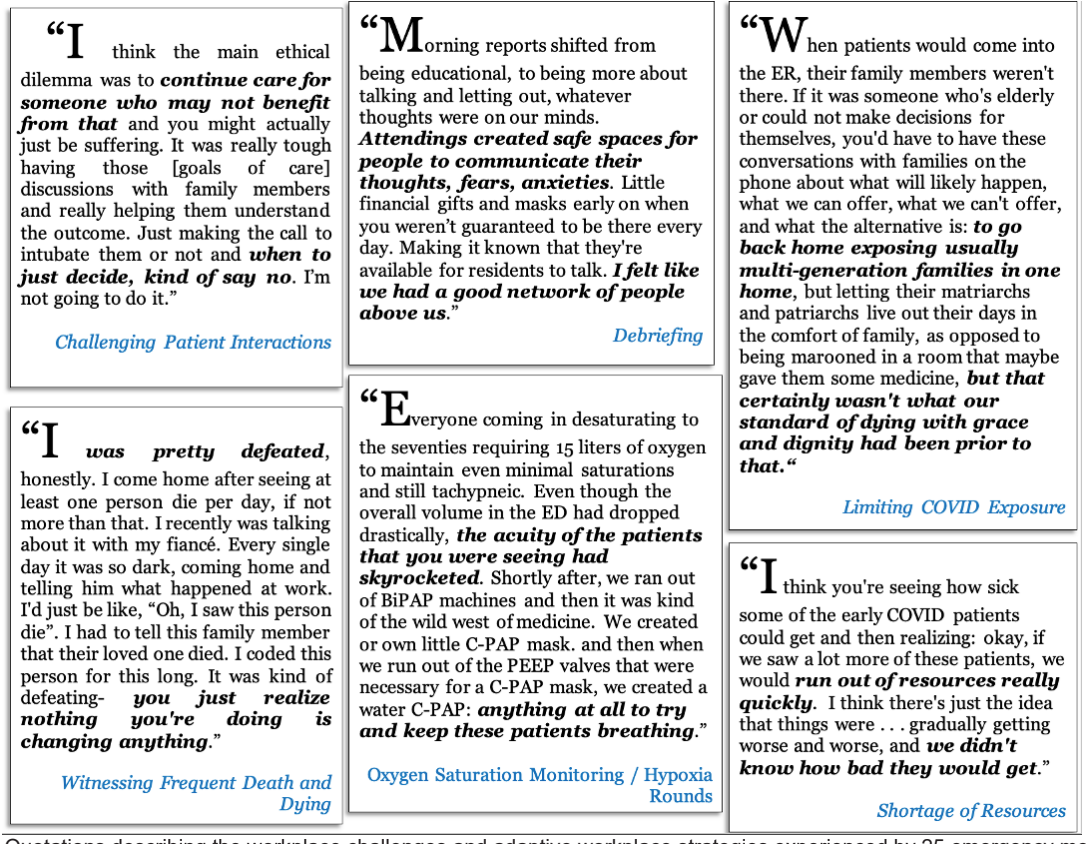
Quotations describing the workplace challenges and adaptive workplace strategies experienced by 25 emergency medicine resident physicians when working and training during the first wave of the COVID-19 pandemic in two urban US hospitals, 2020. *COVID-19*, coronavirus disease 2019; *US*, United States.

Emergency medicine residents were faced with the following workplace-related challenges: difficult patient and family discussions; limited knowledge surrounding COVID-19 pathophysiology and evolving recommendations and protocols; higher volume of patients arriving in the ED with more severe acuity; challenges to transparency of administrative decisions; shortage of hospital resources, including supplies and staff, further exacerbated by the pandemic; witnessing frequent death and dying patients; managing admitted or boarding patients in the ED; social determinants of health; patient presentations limited to COVID-19 and its complications; death and illness of colleagues as well as illness of the interviewee; transitioning from the work to the home environment. (See Figure 3 for a diagram of these themes and Figure 4 for a sample of quotes in these categories.) A complete codebook of themes, definitions, and representative quotes can be found in the Supplemental Materials. Of note, the order and layout of the ranges of responses in Figures 1 and 3 are not arranged with a particular hierarchy but rather are meant to graphically display all major themes found in at least 10% of interviews.

Residents recalled several approaches that the residency program, ED, and hospital administrators took to address workplace challenges, including the following: limiting Covid-19 exposure through a no-visitor policy; establishing hot and cold zones in the ED; policies initially barring residents from participating in aerosolizing procedures; and personal protective equipment (PPE) protocols. Other workplace strategies included city-level, hospital-level, and physician-level decisions not to intubate certain patients or perform cardiopulmonary resuscitation because of medical futility or risk to staff. One of the hospital’s responses to increases in patient volume and acuity in the setting of a worsening shortage of resources was to increase staffing through Federal Emergency Management Agency and US military healthcare workers.

Other department- and residency-level policy implementations included intentional visibility and communication availability with ED administrators, formal and informal debriefing sessions, enforced breaks and days off, procuring tablets and other devices to facilitate family and patient conversations, and residency didactics via the Zoom virtual meeting digital platform. Finally, residents and attending physicians designed and implemented hourly oxygen saturation monitoring rounds, while attending physicians worked with ED administrators to streamline and discharge hundreds of ambulatory, non-hypoxic patients from triage or the waiting room.

### Resident-Focused Wellness Activities

While some residents mentioned residency specific activities created for the purpose of resident emotional well-being during this period without prompting, many residents responded to questions that asked them to specifically comment on resident-focused wellness activities or quality improvement interventions, resident education, as well as offered advice they might give to other residency programs. Emergency medicine residents were appreciative of wellness activities and found them to be a source of emotional support. Specifically, they felt supported by the community and appreciative or proud of their co-resident colleagues for procuring meals, PPE, gift bags, and other donations. They appreciated Zoom hangout sessions with their co-residents and scheduled days off.

### Resident Training and Education

Residents recalled their training and education during this period. They mostly cited experiential or self-directed learning. They also mentioned limitations in their training by the challenges of lack of patient presentation variety, with most of their patients having some degree of COVID-19-induced hypoxic respiratory failure or COVID-19-induced diabetic ketoacidosis. Although never formally coded, a few residents did mention that this monotony had affected their overall training, but that perhaps the benefits of working during a pandemic outweighed the shortcomings, and that they felt that they would fill this knowledge gap either during the remainder of their residency or afterward. Residents reported learning from their co-residents, including bedside training on how to make an innovative medical device using existing hospital supplies such as ventilator tubing, a bilevel positive airway pressure mask, a viral filter, and a positive end-expiratory pressure (PEEP) valve or a canister of water, for the purpose of providing PEEP during periods when respiratory and oxygenation-assisting devices were lacking in the hospital.

Junior resident oversight and bedside teaching by senior residents and attending physicians was a challenging aspect of resident education, due to limited knowledge and evolving protocols surrounding COVID-19 and a higher volume of higher acuity patients. However, junior residents felt supported by their senior residents and attending physicians who saw a large volume of patients and offered emotional debriefing, rather than focusing specifically on bedside teaching. Education and emotional well-being were promoted by Wednesday morning didactic conferences and daily morning report, which were eventually moved to the Zoom virtual meeting software platform and sometimes included resident-initiated group talk therapy and reflection.

## DISCUSSION

### Key Findings

A major objective of this exercise was to develop a comprehensive narrative of a prolonged traumatic shared experience faced by EM residents during the dates recalled as the first wave of the COVID-19 pandemic at two urban hospitals in a US epicenter. The purpose was to provide future EM residency program leadership and residents with this insight to prepare for and manage similar future unexpected pandemics or other prolonged disasters and mass triage events. Key informant interviews took place 3–4 months following this period and EM residents recalled and discussed several major aspects of their experience working and training during these dates of peak patient volume and acuity. Interview questions focused on the general experience, but also specifically on education, interpersonal relationships, and resident-focused interventions within the training program. Major themes emerged: 1) EM residents recalled several workplace challenges; 2) adaptive workplace strategies to address these challenges, as well as their own 3) challenging or 4) protective interpretation and emotional response to these challenges.

In summary, this was a very complex and unanticipated situation for these EM residents as they faced the uncertain morbidity and mortality stemming from the COVID-19 illness, at a time when there was limited knowledge of its pathophysiology, method and likelihood of transmission, patient risk factors, and predicted duration of the pandemic. Emergency medicine residents working at these urban hospitals in a COVID-19 pandemic epicenter during the initial peak volume and acuity dates of the COVID-19 pandemic knowingly risked their lives as they watched countless patients and some of their own colleagues, including attending physicians, nurses, and patient care technicians, become ill and die. They had countless difficult patient and family conversations, and they made ethical decisions brought about by medical supply and staff shortages in a healthcare system that was quickly and unexpectedly overwhelmed by a high volume of sick patients. Residents were often unaware of reasoning and considerations behind administrative actions and behind local, state, and national public health policies, often receiving conflicting messages.

Prior to the pandemic, residents had already been addressing disease outcome inequities caused by social determinants of health, including chronic staffing challenges already existing in these hospital systems. With the increased patient volume and acuity during these COVID-19 peak dates, they also faced an acute shortage of oxygen canisters, ventilators, PEEP devices, high-flow nasal cannula machines, negative pressure rooms, general bed capacity, and PPE. Residents inevitably had to manage admitted patients on behalf of overwhelmed inpatient teams. Their participation in medical education was limited by a lack of variety of patient presentations, and they had difficulty transitioning from their work environment to their home environment.

Some of these challenges were addressed by administrative policies and resident wellness activities. Despite these interventions, EM residents faced difficult emotions: they felt exceedingly fearful, anxious, sad, overwhelmed and powerless, unappreciated or undervalued, lonely and isolated, and burnt out, and often demonstrated post-traumatic stress responses. However, with the help of workplace and outside of work emotional support, residents were able to adapt and display evidence of their emotional resilience, appreciate their colleagues and community support, and persistently show empathy for their patients. They found ways to feel useful, were hopeful, and accepted aspects of a new reality. They felt inspired by and supported by their workplace teammates, were proud of their work, and maintained a sense of duty to provide patient care to the best of their ability.

### Comparison To Previous Research

There have been several perspective and commentary pieces written by training program leadership outlining the measures taken during a disaster period to address residency training challenges.^12^–^14^ They comment on the need for providing clear communication from leadership, establishing resident wellness committees, and guaranteeing PPE and the measures taken to do so. A commentary by chief residents of a medical training program on adaptive strategies applied at work highlights the application of scheduled updates and communication from residency leadership, and the creation of a space for debriefing and maintaining emotional connections between coworkers through online conferencing.^14^ One participatory observational study looking at residents’ perceptions of their education during the COVID-19 pandemic found that residents felt their didactic education time and their attendings’ involvement in formal education decreased.^15^

Our study is the first to analyze the emotional and workplace challenges and perceptions along with the adaptive and protective strategies employed by postgraduate medical trainees and the training program in a pandemic or disaster period. While these previously cited papers individually recollect what interventions were undertaken by residency leadership, ours delves into the breadth of the workplace *and* emotional challenges that were encountered. Our study also provides participant-informed feedback on implemented adaptive strategies, experienced protective factors, and suggestions for future pandemic and disaster response scenarios.

## LIMITATIONS

Specific approaches were taken to reduce some of the limitations commonly found in qualitative analyses. Measures were taken to have a diverse pool of participants; however, accuracy of participant representation of each class and the entire residency program was not measured quantitatively but rather subjectively by all authors. (See Table 1 for some diversity data.) A potential limitation was that all residents worked in an urban setting; therefore they were not necessarily representative of rural or suburban communities. Further, the accuracy and relevance of some perspectives may vary based on the participant resident’s level of training and their likelihood of having acquired knowledge and experiences such as coping mechanisms and comfort with end-of-life discussions.

While residents were asked during their interview not to share the content of interviews with any other resident, this may have still occurred, and may have potentially biased some responses. While confidentiality was agreed upon prior to interviews, there may have been some hesitation for informants to speak with complete candor for an interview discussing their experience, possibly limited by a perception that administrators may not have wanted them to be completely transparent surrounding perceived challenges or failures. In a complex traumatic event, the experiences of residents are expectedly iunique, dynamic, time-limited, and subject to memory-related biases. The willingness to partake in a qualitative interview with the primary investigators may potentially correlate positively with satisfaction surrounding administrative interventions related to COVID-19, although only one recruited EM resident refused to participate. Participants may have felt compelled to participate given their knowledge that senior EM residents and an EM graduate medical education administrator formed part of the research team.

While theme saturation was achieved for the thematic categories, only 25 residents were sampled; therefore, identified themes may not be an exhaustive list of perceptions an EM resident may experience when faced with a prolonged disaster environment. While final theme creation and selection may have been biased by investigators’ membership within and shared experiences with the group under study, we believe these study outcomes represent a sufficiently broad and nearly comprehensive range of possible experiences and perceptions.

The dates we focused on were chosen by our PIs, who were participant observers in relation to the population under study, with a level of complete participation in activities of the group under study, also having worked clinically in these EDs alongside the participants during the focus dates and prior to the initiation of the interviews. There was a consensus among the investigators that the most noticeably challenging dates for COVID-19 at our hospitals, coinciding with the highest number of high-acuity COVID-19 patients compared to other dates, were March 15–April 11, 2020. Therefore, the interviewers repeatedly focused and refocused interview questions on these dates. However, it is possible that focusing on a longer period or asking generally about experiences during the first pandemic waves may have had the potential to yield a more comprehensive exploration of residents’ experiences related to the disaster and mass triage period under study.

## CONCLUSION

This study demonstrated that EM residents have a noteworthy perspective as key frontline hospital responders during a prolonged disaster and mass triage event within a local healthcare system. For example, many residents mentioned that there were informally enforced breaks with food delivery and how this created a space to step away from the clinical area as well as spend time to talk to and receive emotional support from colleagues. Further studies to examine the effect of enforced breaks on wellness/emotional well-being may be indicated from these findings.

Furthermore, residents mentioned positive interactions with administrators during daily briefings. Further study is indicated to see the benefits, if any, of formal briefing as a policy. Key decision-makers in health system administration and emergency preparedness should consider protocolization of treatment plans and conversations regarding end of life. Implementing supervision quality checks of these difficult do not resuscitate/do not intubate conversations may allow junior residents real-time feedback. Asking supervisors to enforce and encourage breaks during working shifts, having a formalized and enforced PPE policy, as well as having readily available or on-site access to mental health resources may improve resident wellness and wellbeing and increase productivity.

While these findings can be applied broadly to other training programs and other disasters and prolonged mass casualty events, including those outside the United States and outside of emergency medicine, more quantitative and qualitative research in other sites as well as in the context of other pandemics is needed to establish these findings as universally applicable.

Since the start of the COVID-19 pandemic, there have been fluctuations in cases and new variants of COVID-19. It is anticipated that the world will face more infectious disease pandemics in the future. Most of the global population remains unvaccinated against COVID-19. Therefore, it is important for key decision-makers in resident education, hospital administration, and all levels of public health management, to inform themselves about residents’ emotional and workplace challenges when establishing hospital and residency program disaster protocols. We suggest that the frontline resident experience should be prioritized accordingly in any healthcare system’s response to unexpected pandemics and disasters, as providing emotional and material support to residents is likely to help residents be more effective in the workplace. More research is necessary to determine whether these interventions can prevent the long-lasting negative psychological effects of facing a prolonged trauma in and out of the workplace.

## Supplementary Information



## Figures and Tables

**Table 1:** Respondent demographics

Demographic	Percentage (%)
Gender	
Female	13/25 (52%)
Male	12/25 (48%)
Age	
27–30	11/25 (44%)
31–34	11/25 (44%)
35+	3/25 (12%)
Relationship status	
Single	6/25 (24%)
In a relationship	19/25 (76%)
Living situation	
Alone	4/25 (16%)
With others	21/25 (84%)
Ethnicity	
African American	3/25 (12%)
Asian	4/25 (16%)
Caribbean	1/25 (4%)
White	9/25 (36%)
Latino	1/25 (4%)
South Asian	4/25 (16%)
Middle Eastern / North African	1/25 (4%)
Mixed (including AA/Latino, Asian/AA)	2/25 (8%)
PGY Level	
1	6/25 (24%)
2	6/25 (24%)
3	6/25 (24%)
4	6/25 (24%)
5	1/25 (4%)
Program	
Emergency Medicine (EM) Categorical	20/25 (80%)
EM/Internal Medicine (IM) Combined	5/25 (20%)
Sick with COVID	
Yes	13/25 (52%)
No	12/25 (48%)

*COVID*, coronavirus disease 2019.

## References

[1] Grennan D (2019). What is a pandemic?. JAMA.

[2] New York City Department of Health and Mental Hygiene (DOHMH) COVID-19 Response Team (2020). Preliminary Estimate of Excess Mortality During the COVID-19 Outbreak - New York City, March 11–May 2, 2020. MMWR Morb Mortal Wkly Rep.

[3] Thompson CN, Baumgartner J, Pichardo C (2020). COVID-19 outbreak - New York City, February 29–June 1, 2020. MMWR Morb Mortal Wkly Rep.

[4] Brooks SK, Dunn R, Amlôt R (2019). Protecting the psychological wellbeing of staff exposed to disaster or emergency at work: a qualitative study. BMC Psychol.

[5] Morris AM, Ricci KA, Griffin AR (2016). Personal and professional challenges confronted by hospital staff following hurricane sandy: a qualitative assessment of management perspectives. BMC Emerg Med.

[6] Imai H, Matsuishi K, Ito A (2010). Factors associated with motivation and hesitation to work among health professionals during a public crisis: a cross sectional study of hospital workers in Japan during the pandemic (H1N1) 2009. BMC Public Health.

[7] Raven J, Wurie H, Witter S (2018). Health workers’ experiences of coping with the Ebola epidemic in Sierra Leone’s health system: a qualitative study. BMC Health Serv Res.

[8] Ives J, Greenfield S, Parry JM (2009). Healthcare workers’ attitudes to working during pandemic influenza: a qualitative study. BMC Public Health.

[9] Brooks SK, Dunn R, Amlôt R (2016). Social and occupational factors associated with psychological distress and disorder among disaster responders: a systematic review. BMC Psychol.

[10] Tremblay S, Castiglione S, Audet L-A (2021). Conducting qualitative research to respond to COVID-19 challenges: reflections for the present and beyond. Int J Qual Methods.

[11] Sofaer S (1999). Qualitative methods: what are they and why use them?. Health Serv Res.

[12] Kemp MT, Rivard SJ, Anderson S (2021). Trainee wellness and safety in the context of COVID-19: the experience of one institution. Acad Med.

[13] Newman B, Gallion C (2019). Hurricane Harvey: firsthand perspectives for disaster preparedness in graduate medical education. Acad Med.

[14] Ostapenko A, McPeck S, Liechty S (2020). Has COVID-19 hurt resident education? A network-wide resident survey on education and experience during the pandemic. J Med Educ Curric Dev.

[15] Rakowsky S, Flashner BM, Doolin J (2020). Five questions for residency leadership in the time of COVID-19: reflections of chief medical residents from an internal medicine program. Acad Med.

