# Supplementary material for: Emergency Medicine Residents’ Perceptions of Working and Training in a Pandemic Epicenter: A Qualitative Analysis
Source: West J Emerg Med. 2022 Dec 30;24(2):269–78. doi: 10.5811/westjem.2022.9.57298 (PMC10047728; doi:10.5811/westjem.2022.9.57298)
Supplement: Supplementary file 1 [file wjem-24-269-s001.docx]

1. SUPPLEMENTAL MATERIALS
2. Supplemental 1. WORKPLACE CHALLENGES

| **Theme** | **Definition** | **Example** |
| --- | --- | --- |
| Challenging Patient and/or Family Interactions | Challenging conversations with patients or patient families, including providing reassurance and delivering bad news | PGY3: It was tough, they all happened over the phone, obviously, families were very distraught; and the worst parts were always that- they would ask to see if they could see their loved one before anything may happen to them. And just saying to them: I'm sorry, I really wish I could, but you just can't right  now- we can't put people at risk in the hospital at this moment. That was really hard. During the peak of COVID, one of our attendings would be post-shift, just writing notes and he would get a call at the ER. And there'd be a family member saying- Hey, I'm looking for so and so, do you know if you can help me and try to find them and he would look them up and he'd say: I'm so sorry, your family member actually passed away a day ago, two days ago. And he would say: did you know, and the family member would say: no, I had no idea. So the attending would have to console them through that. So obviously that's a very horrible experience. |
| Limitations in Knowledge or Best Practices and Evolving Protocols for COVID-19 | General lack of knowledge in treating patients with COVID-19, changing workplace protocols, and self-guided learning with limited published or peer-reviewed data | PGY4: We’re used to being able to change the trajectories of critically ill patients in most cases. Or at least know that we can make a big impact and we can provide comfort care and support our patients if we don't feel like we can change their trajectory of illness. But we were seeing a lot of relatively young and healthy people who were critically ill. And we didn't know if any interventions we were doing were truly helping. And we were getting conflicting information about what to do, like intubate early, don't intubate early; do noninvasive ventilation, don't do non-invasive ventilation. It was all confusing and we were trying out different things, but there've been very few situations where we clinicians have been put into a position where we didn't have more clear information or have a stronger knowledge base on what actually works and what the right thing to do for patients is. And we were seeing the same thing over and over again and running into the same issues and it made it even more frustrating because it wasn't like this was the first time I saw someone who was in respiratory distress and hypoxic from COVID. Every time, we didn't know what to do and we just tried to do our best for them. |
| Higher Volume of Patients with More Severe Acuity | Overcrowding with a higher level of acuity, higher acuity patients being cared for in lower acuity areas, or Pediatrics ED, as well as mass triaging and discharge from the waiting room. | PGY5: I remember an Attending said: you're a duck in the water and then you're just trying to move. But you don't see how fast your little feet are swimming in the water. And so that's how it was for your shift: you're just so busy. But even then, this was on a different level because you're normally not dealing with as many sick people as we were dealing with, and then everybody's sick and everybody in the ER is sick and everybody is having breathing |

|  |  | problems and then you're seeing numbers you've never seen before. And you're just trying to make sure nobody dies on you . . . basically, you're just trying to do the bare minimum while trying to keep everybody alive and safe as possible. |
| --- | --- | --- |
| Shortage of Resources | Difficulty in the timely acquisition of PPE, hospital equipment, staff, limited availability of regular hospital services, limited in-person consultants, and supplemental oxygen tanks | PGY3: I think you're seeing how sick some of the early COVID patients could get and then realizing- okay, if we saw a lot more of these, our resources would run out really quickly. There's just the idea that things were gradually getting worse and worse, and we didn't know how bad they would get.  It was definitely different, just with how many sick patients there were. In the way we were thinking, it was almost like the way you think during a mass casualty incident- really triaging people. I think there are a lot of patients who probably didn't get treated with the standard of care or like normal time because we were triaging our resources towards the patients where we thought they would get the max benefit. |
| Witnessing Frequent Death or Dying | Frequently witnessing death and rapid decompensation of patients in the ED | PGY4: You know, when you are doing what the book tells you... and, it doesn't necessarily help  . . . or it helps and then they die two days later and you're just like, “what is going on? This is such garbage” ...I think a huge thing that was affecting people was the people that were boarding in the ER for so long. You can have critical patients that probably have a poor prognosis. You send them to the ICU and you're not necessarily watching them die, like over the course of a few days. But in the ED, when you're there, managing these people for several days and they die in the ED, it's a side that we don't really see a lot. And I think a lot of ED docs get some element of depression in ICU settings because we are used to having those quick victories and it does possibly  secure a false sense of accomplishment for us. And that veil was lifted for sure. |
| Managing Admitted Patients | ED residents placing orders for, having conversations regarding, or otherwise clinically managing patients who have already been admitted to the hospital but are boarding in the ED while they are waiting to be transported from the ED to another assigned area of the hospital, such as the Med-Surg units or the ICUs | PGY2: It was a very different experience trying to manage these patients, and by that time we had more nursing support, but making sure all of the labs were repeated. I was reminding the nurses to repeat the labs as I do hypoxia rounds and remind people to repeat labs. It was a weird juggle that I felt uncomfortable with because I didn't feel like I had a handle on it like I do my ED patients. I was worried that I was missing things because the inpatient team had said: just deal with it. And I didn't feel comfortable dealing with their patients when I had my own patients, but we were all in this together. |
| Social Issues in the Community/ Social Determinants of Health | Patients’ and community psychosocial, economic, family hardships | PGY2: There was this ongoing question of: are we doing enough? Are patients all dying because we're not a good enough hospital? If they were in a different hospital, would they be doing better? If we had more nursing, would |

|  |  | they be doing better? Is it us? Is it the residents? Is it the doctors? Are we not doing a good enough job? So there’s that question that gnaws at you. Did it just happen to be that our community baseline is so sick and then they are doomed to this fate regardless of where they are, who's taking care of them?  You look over at Manhattan and they're doing a lot better, so you constantly wonder- you know, is it you, could you be better? Could you have done something better for them? And I don't know if we'll be able to answer that. And now that COVID is happening in other low socio-economic status environments, I'm sure they're also wondering the same thing. "Why are our poor communities doing so much worse?" |
| --- | --- | --- |
| Monotony/ Lack of Variety in Patient Presentations/ Patient Illness | Almost exclusively seeing COVID patients with respiratory illness in the ED | PGY2: There was this ongoing question of: are we doing enough? Are patients all dying because we're not a good enough hospital? If they were in a different hospital, would they be doing better? If we had more nursing, would they be doing better? Is it us? Is it the residents? Is it the doctors? Are we not doing a good enough job? So there’s that question that gnaws at you. Did it just happen to be that our community baseline is so sick and then they are doomed to this fate regardless of where they are, who's taking care of them?  You look over at Manhattan and they're doing a lot better, so you constantly wonder- you know, is it you, could you be better? Could you have done something better for them? And I don't know if we'll be able to answer that. And now that COVID is happening in other low socio-economic status environments, I'm sure they're also wondering the same thing. "Why are our poor communities doing so much worse?" |
| Death/Illness of Staff/ Faculty | COVID illness of faculty/staff/coworkers/or self, or death hospital staff due to COVID | PGY2: I remember when I first saw my heart rate and o2 sat and I kind of freaked out because it's when people were first starting to get intubated and stuff in the ED. I made a contingency plan for myself. I tried to figure out which ER I would go to, based on my location. I remember actually, the chief on call...and other residents were checking on me- every couple of hours they would text me to see how I was, which was amazing. Some of our classmates too, someone made me check my pulse ox every four hours and report it back to her. I was very aware of the risks. It freaked me out mostly that my heart rate was 135 and it obviously was that high when I'm exercising but sitting there at rest, I don't think I've ever had a heart rate that high. It freaked me out. |
| Transition to Home | Leaving work, changing routines to attempt to limit exposure/keep their home a “cold zone” | PGY2: So it is just the main thing is you don't want to bring home COVID. Timing things, changing before you even head out, changing |

|  |  | into new scrubs or new clothing, going into the train, watching around to see if there are any people looking at you funny and if you feel they're particularly aggressive. I'm going home and then changing immediately when I got home and then going into the shower. So there's this whole long process of just trying to maintain this invisible infection that you're not even sure what you're supposed to do. So it’s a lot of maintaining the unknown. Every day, it would be just door to door, changing everything. The moment we got home, shower before anything is done and finally, then you can eat dinner and relax and whatnot. It was a long process. |
| --- | --- | --- |

3

4 Supplemental 2. ADAPTIVE WORKPLACE STRATEGIES

| **Theme** | **Definition** | **Example** |
| --- | --- | --- |
| Limiting COVID Exposure | Family member exclusion from ED, Hot/Cold Zones, initial policies of not having residents seeing COVID patients, initial policies barring residents and then junior residents from aerosolizing procedures including intubating, limiting COVID exposure through the use of PPE | PGY1: For engineering controls, with [the] state's banning meetings, we didn't have any more in-person meetings for conferences. We made sure all the PPE was stored in one place. But then we had temperature checks by the entrances. . . The security guard or the triage nurse made sure everyone was wearing the appropriate PPE before they walked inside the emergency room- that was a big thing. |
| Decision Not to Escalate Care | Discussions had or decisions made at all levels to not escalate care for specific patients given poor likelihood of improved morbidity and/or possible harm to the patient | PGY3: Obviously everyone is worried about running out of ventilators. I think because of that, because there was this idea that we have a scarce resource, people were much more inclined to push patients, like really sick patients, to be DNR/ DNI, figuring: “okay, if they, if we intubate them, they're just going to die on a ventilator and use it up for someone else...” At the time it did seem like there's this really real possibility that we were going to actually run out of ventilators. I feel sort of bad because it's our patient population; the scarcity likely affected how we took care of our patients, but maybe not. I don't think people would have acted the same way at a hospital with more resources and a patient population with more resources.  Patients in our patient population are very deferential to physicians. I don't know if we like took advantage of that, but I do feel like there was a strong, paternalistic sense of: “you should really be made DNR DNI.”, I think a lot of the patients probably would have ended up dying on ventilators, but I think in some ways people pushed a little bit too hard saying that there was no hope. |
| Staffing, Surge, and Flexibility | Surge call residents and ED resident flexibility in the hospital allowing for resident days off; temporary ED staff through FEMA, the US military branches, or other per diem contracts | PGY4: I think the thing that stood out to me the most was how much everybody wanted to do everything and help everything at all times. I'll never forget [this resident]. I think on probably my worst day when I was down in the ER, she's just like: what do you need? Is it drawing blood? Is it doing everything else?  And there were multiple other residents who would just do the same. |

|  |  | The amount of ingenuity from our co- residents, and a willingness to do whatever it takes to make it work and work together and support each other, however it was needed, whether you needed to take time, whether you needed to do other stuff. I think it didn't surprise me at all but in the same sense, it was very reassuring about how well we worked together. |
| --- | --- | --- |
| Visibility and Communication with ED Administrators | Positive or improved interactions with hospital administrators, including frequent updates, emails, and in-person appearances for the purpose of communication about protocol updates and supply status | PGY4: I think our direct administrators and our attendings did a really good job at trying their best to communicate with us while they're dealing with a lot of external forces on them. I think they did the best they could in the situation they were presented with. And I think it was really hard to predict the trajectory of what was going to happen. And I feel like maybe people in the upper levels of government, maybe like higher levels in our own admin didn't perceive what was actually happening on the ground and that made their job even harder. I guess that's kind of convoluted, but I think they did a good  job and I think they had a lot of pressure in a lot of different ways that made their job very difficult. And I think they were  working incredibly hard and were incredibly responsive and, even though they were very overwhelmed with everything they were doing, if there was ever a situation where me or one of my co residents wanted to talk directly to the highest levels of our administration, they were always ready to hear from us and talk to us. And for people that were struggling or people that were especially worried or needed a break, they were given that time and I think that was really good for certain people... I think the fact that administration started coming to our conferences and giving us real time updates and also answering all our questions was possibly the best thing in terms of the best intervention we had |
| Debriefing | Senior residents debriefing with Junior residents, staff creating a space to talk about feelings, attendings asking residents about their well-being given the context. This also includes Wednesday Simulation Sessions dedicated to debriefing | PGY1: It was nice to speak to other people and know that like I'm not alone in, um, the feelings that I'm having, you know, like you can feel, you can definitely feel like-, why am I not feeling this kind of thing, you know, situation and knowing that everyone else had the same feeling of numbness and then slowly realizing what had happened - that was comforting... You know, usually you don't have time to step away to have a meal and this kind of forced everyone to take time to 1) step away and take a meal, but also to talk to each other, which was nice. . . I think there's- multiple people in our residency that are good about talking about their feelings. And I think they made a point of pushing all of us to process what was going on. |
| Enforced Breaks | Attending/co-resident encouragement to leave the clinical area to take a break and eat outside of the clinical area, including when food was donated. This also includes when residents | PGY3: I think that the attendings that we work with fortunately recognize how difficult this was for everybody, for the residents. And they knew: Hey, if you need a break at all, |

|  | were dismissed early | please go ahead and take a break. If you need a shift off even- go ahead and take a shift off. If you need me to see these patients, I'll go ahead and do that. They made sure that we were well fed, that we were feeling okay at home. They'd ask how our families were doing. I remember, when I was actually working, my wife had gotten sick, so I was pretty concerned during that time and the administration and chiefs were asking me every day, how she was doing, making sure she was okay. And obviously making sure that I was okay. So I really appreciated that. I'm sure that it wasn't just me. I'm sure they did that for anybody who is experiencing that situation within our residency. |
| --- | --- | --- |
| Technology to Facilitate Family Conversations | Electronic tablets for family conversations and patient communication with family outside the hospital. Also includes when residents and staff used their own phones for patients to communicate with their family members | PGY4: But obviously New York was very unique and being one of the first places to completely shut out all visitors, completely shut down everything. and, although it was difficult and slow, the amount of effort and things that people did, and I think this probably goes with the QI thing that you mentioned was, how much effort people did to try to relieve that problem. Whether it was setting up iPads to allow people to do it. Most people ended up just getting on their phones and FaceTiming people for ease of things or when something was an emergency and other stuff, and also realizing that: I don't know how people are going to experience it in other places. And it's probably, at least the way that I read about media and everything- things are going to be different. But really thinking about and anticipating dealing with families and issues, especially for the more sick and severe patients of how you're going to cover it. I think there were a lot of institutions that stepped up in New York to do things like that. Whether it was providing- in a hospital with very limited resources , even we were able to come up with ways to allow people to communicate and make decisions and things a little bit easier and still able to work. |
| Zoom Conference | Virtual Wednesday didactics conference and virtual daily morning report | PGY3: I'm all about the *Zoom* conference. It was good. It was nice to at least be able to hear everyone's voices, even though I couldn't see them in person. I did miss seeing everyone at an in-person conference. That was something to look forward to. But yeah, it was nice to kind of have a semblance of normalcy during all of it. And then I really liked when, I don't know if this was during the end of March early April, but when [the program director] started doing his 15 minute good news spiel at the beginning of every conference, it was nice to hear and always nice to see someone- like here's some positive news. I liked having conferences and hearing about something that wasn't COVID related and being able to still hear the voices of my co-residents. |
| Oxygen Saturation Monitoring/Hypoxia Rounds | Providers began to incorporate “hypoxia” rounds, which were frequent and periodic, portable pulse-oximetry of patients (who were | PGY1: You would come to shifts...even try to get there a little bit early at that point, I think, to start hypoxia rounds while people were |

|  | often in the hallway on oxygen tanks), providers would titrate supplemental oxygen as needed/replaced oxygen tanks as needed and then document for other providers to follow. Also includes when the interviewee mentions creating makeshift positive pressure masks and connecting to oxygen supply | finishing up things from the last shift, and basically you're just taking care of patients that are already admitted. And you turned the ED area into a mini ICU or actually a bigger ICU, to be honest... and constantly trying to think of little ways to help people not die. So someone came up with the brilliant idea of printing out the oxygen sheets, what time, how much oxygen they were on, what their saturation is and if any changes were made.  And so then you just go around and try to find any beds that didn't have that taped on them, tape that on them. Then trying to make sure you found all the peep valves that you could to hoard them for the shift or you pre-made some of those C-PAP things so that when you actually needed them, you weren't running around trying to find everything, trying to see how much oxygen you have left for the night, because we ran out of oxygen tanks. |
| --- | --- | --- |
| Waiting Room Triage | Attending physicians seeing ambulatory non- hypoxic patients in the waiting room/triage to assess them for potential discharge home with followup | PGY2: When this particular adult attending was on, she would go out into the peds waiting room, which got transformed into an adult triage before the waiting room area...and she would just triage everybody, hundreds of people before anyone ever even got to the waiting room area, just any well patients. And that was so commendable. I don't think many people knew that this was happening or the volume in the ED would have been easily two to three times higher. |

5

6

7 Supplemental 3. EMOTIONAL CHALLENGES

| **Theme** | **Definition** | **Example** |
| --- | --- | --- |
| Fear and/or Anxiety | Feeling fear, and anxiety surrounding broad and specific situations, including acute stress about the transmission and severity of the virus and provider limitations, fear of the unknown, difficulty obtaining PPE | PGY2: I was scared of the unknown and scared of what I already did know. Largely for the patients, but also our older coworkers, attendings, all that stuff. People who are still working that, you know, didn't expect to be working still. I had already gotten COVID by that time and I knew how uncomfortable it was. I kind of figured that, given the statistics on age and everything, there were some people that I was worried for who were still working. And I think seeing everybody in the ED made that more real too, that they were still working and it freaked me out. |
| Frustration and/or Anger | Feeling frustration or anger surrounding broad and specific situations | PGY4: And when I look now to all these hot pockets in the United States, like Florida and Texas and California, and it's August, or where are we now? Yeah, we're in August. And we're in August and our peak was in March and April. And you see these peaks where their ICUs are full there and their hospital beds are full and they're running out of PPE. Part of me is angry because it's been so long and we were so vocal and rang the bells so loudly and how bad this thing is. And so all that time feels a little wasted and all these patients who are dying unnecessarily, that feels very much in vain. Our efforts in our New York Times articles and our Facebook posts and all these |

|  |  | things kind of are falling on deaf ears...I have to imagine it's because it is so novel and it's so unique in its presentation and how it manifests that there's no way that we could have done any more until they've seen it with their own eyes until you've been touched yourself, which is terrible, you can't really appreciate how hopeless and how terrible this virus manifests in real life. So, well, it's annoying. I kind of get it. Who could have described what we saw in March to us. Before it hit us. We wouldn't have believed them. So somebody would have said here's patients, they have vital signs that are incompatible with life. There were zombies. They can't even give you their names. And you're like, that sounds weird as hell, but then we saw it and then it was our whole reality. And if you try to explain that to somebody you say, I took care of his zombie today, they're going to be like, Oh, that sounds weird. There's no way to really relay that kind of experience. |
| --- | --- | --- |
| Disrespected and/ or Dispensable | Feeling disrespected, dispensable, undervalued, or dehumanized because of lack of communication, transparency, organizational and governmental support, recognition, or hazard pay | PGY1: A lot of the communication that was happening was not happening between residents and administration. It was happening between administration and chiefs. And even the fact that communication was happening was not communicated to the residents. So it felt like we were in the ED working 12 hour shifts going home after that, sleeping a couple hours, going back to work; things weren't working and nobody cared. Of course, people did care because people were talking about it behind the scenes. But when you don't know that people are talking about it, you feel abandoned and you don't feel like you're a part of the process when actually you're the integral person in the process. So being like a worker bee, who is exposing themselves to all of these, to the virus where we don't know a lot about it, Most of us ended up getting it who were working during that period and not actually having a say in the decision making process or even being able to give feedback in a meaningful way. I felt like we were very disposable and I was being treated like a child. Frankly, a lot of us have had lives before working in medicine. A lot of us have a lot of expertise prior to working in medicine. Right? And the fact that some of the administration, who some of these leaders have had no careers outside of what they've been doing, are now trying to figure out workflow design, engineering problems and the fact that they're not reaching out and tapping on residents who were willing and able to give extra hours after they were working to get ideas. That felt very, I'm not sure what the motivation was behind it from their side, but, it just felt like we were wasting a lot of manpower. |
| Unprepared, Overwhelmed and/or Powerless | Feeling unprepared, feeling self-doubt, feeling clinically inexperienced, feeling imposter syndrome, feeling powerless, feeling inadequate, feeling helpless, feeling ill- equipped, feeling overwhelmed, or feeling ineffective at work. This includes questioning | PGY4: Compare? Comparing apples and oranges. Business as usual before, and then kind of the months leading up. There was this kind of heightened sense of it's coming, it's coming. The tsunami is coming. But never really knowing when. And then it hitting was |

|  | the effectiveness of our available treatments and resources or feeling unable to provide the best care to patients because of social issues or shortages. This also includes feeling surprised or shocked about rapid progression or the abrupt onset of the pandemic in NYC, or any mention of an unanticipated course of the COVID-19 pandemic | just from one day to the next it was...yeah..I remember specifically being like it's coming. And then the next day we had like, within an hour, five ambulances coming in with patients who are in respiratory distress. So it really happened from one day to the next day, it was as if all the patients at [omitted] had reached their critical day 10 on the same day. |
| --- | --- | --- |
| COVID-19 as all-encompassing, inescapable, and/or intrusive | Feeling that COVID is all-encompassing and that they are unable to escape it, both in and out of the hospital, or that it is always on their minds, thoughts, and/or in conversations | PGY1: It follows you, it follows you, you know, I come home and I am no longer able to forget about a patient, you know that I saw dying in front of me because like I said, there were so many of them, there were multiple of them.  And when I came home, especially the first few days, all I did was just think about my patients. I've had several young people die, people my age, people who maybe just have a little bit of asthma, maybe a little bit of high blood pressure, die in front of me. And to me, it's like, you know, this could have been me.  You know these are people who are going about their lives just like I am. And all of a sudden, now they're dying and there's nothing that I can do about it. So coming home, thinking about that, you know, it was impossible to leave it behind because you come home, you watch TV and it's there. You know, you call parents, or you call your family members and everyone's asking you questions about it, everyone is asking you about your experience, what have you seen, so the repetition, every single time just brings it to life. And like I said, it was just making it more and more difficult every single day. |
| Humble, Undeserving and/or Resistant to Praise | Feeling humble, undeserving, or resistant to praise, including the interviewee’s reaction to being called a hero, 7 pm cheering, food donations/gifts, and other expressions of praise or support | PGY2: I didn't feel like a hero. I'm [crying] I'm sorry- give me a second. I don't think that...I don't think that the patients that I told, that they were going to be okay--and that was the last thing they heard, thought of me as a hero. So I think that was really tough. Seeing that like a lot. Especially in the media or just simple things like coming home and having people clapping at 7:00 PM at change of shift. It just reminds me of how much of a hero that I was...[crying] |
| Loneliness, Isolation, and/or Homesick | Feeling lonely, isolated, abandoned, excluded, or homesick | PGY2: It was lonely. I don't know. It was lonely because well we're people people, I think, especially in the ER, you know, when I look at the reasons I went into emergency med, it had much more to do with the people than anything else. We touch each other, you know, we give each other hugs all the time at work, pats on the back. It's like an elbow to elbow job, where you're in constant communication with the people around you, with your patients. You know, even compared to the inpatient side, I love the part of the ER, where you're just in front of your patients physically, as opposed to being in a computer area and then, you know, separate from your patients and then suddenly, you know, face to face human interactions were gone. You know, in the plans, you know, in the world, erasing our calendars for the next, however many months of the plans that we had, the people we were supposed to see, you know, the things |

|  |  | that we were supposed to do, just all kind of went away. And I think it felt very lonely, both losing our social interactions and our physical interactions. But I think it felt really lonely. |
| --- | --- | --- |
| Sadness or Depression | Feeling sad or depressed, including when the interviewee mentions feeling sad about patients dying or colleague illness and death | PGY1: It made me really sad and sometimes, you know, angry that I wasn't able to do anything. There was one specific case that I remember of someone who was actually one year younger than me who came in, his only medical problem was asthma. Initially he was, talking, you know, he looked like he was in a little bit of respiratory distress, but he was talking to us... became a little bit confused and literally within hours of taking care of him, he codes, we intubated him and everything. When he came into the ER, talking to me and then just a couple of days later to find out that he died, that was one of the many instances where, I will disclose this, that I actually came home and cried. You know, I have  cried several times during COVID, but that was one of the ones that I remember. It also makes you just come to terms with your own mortality, you know, like being young doesn't- it means nothing, especially during COVID times. It literally doesn't mean anything. You can have someone with no medical problems in their twenties, I mean, and die from this disease. It was, it was awful. I was sad. I was angry. Luckily, you know, there were, I had, I have some amazing colleagues, people that I talked to. I have a great support system. I was able to talk to them about it and move on. It's probably going to haunt me for a very long time. |
| Guilt or Remorse | Feeling guilty or remorseful about personal or medical decisions both in and out of work..  This can be in the context of an internal dilemma including a decision regarding patient care or not maintaining established relationships or roles or duties | PGY2: Yeah, it became, like it was ABCDE, call the family. Like, it was just very quick, like, part of it was goals of care, you know, first and foremost, but also just being really clear. You know, once we got to the point that we didn't have ICU beds, we didn't have sedatives, people were not doing well when they were intubated and elderly people were really not doing well. They debated having these phone calls early on in the ED visit about the fact that, you know, your family member’s here, they look like they have COVID, we're going to give pain medicine, we're going to give oxygen, we're not going to intubate because we don't want to, we don't want to contribute to pain and suffering, which was in some ways, probably one of, you know, kind of the worst paternalistic things that we had to do. And in some ways was also in the end, probably one of the more merciful things that we did. It's not prolonged suffering now. I can remember seeing patients on the floor who were intubated, without sedation or getting ICU level needs in floor level care. I don't know if I ever... I'll never know if we did the right thing by these patients. |
| Burnout | Feeling tired, exhausted, apathetic, emotionally detached, or numb, including in the context of lack of patient presentation | PGY4: Dread. Every day. I never ever felt dread going to work for four years, for three and a half years. I love my job and I love what |

|  | variety or learning opportunities | I do, and I love emergency medicine and I love the people that I work with, but there was such a sense of dread when I woke up every morning during those four/five weeks, to like get myself out of that bed and get ready and get myself on my commute and over to the hospital and this overwhelming sense of dread walking up to the ambulance day. And I never, ever, ever felt that before. And I, you know, I'm Latina, I'm late for everything, anything personal I'm late for, but I'm never late for work or at least that is how I have tried to live my professional life and be very punctual when it comes to work. Cause I know how hard it is for me in general. And I overcompensate. But I found myself leaving five, 10 minutes later than I would have arrived right on time or a minute late. And I think it all had to do with this impending sense of dread that I had every day coming to work. |
| --- | --- | --- |
| Post-traumatic Stress/ Secondary Trauma | Feeling lasting effects COVID traumatic experiencing, including triggers for negative or intrusive emotions at the time of the interview or after peak volumes and acuity had diminished | PGY3: Yeah. I mean, I kind of alluded to it earlier, I had never experienced this level of anxiety or fearfulness going into work. I honestly think that it may have lingered a little bit, even for me this many months out. I think I still have some sort of residual anxiety going into work because of the unpredictability of it. And I never experienced that before. I think that's the nature of emergency medicine. And past the first couple months of intern year, I had gotten over that very quickly. But then even now, even after the volume has gone down, even after the acute presentations of COVID have gone down, I still sort of feel that way when I go into work, it's sort of like a dread or a fearfulness. And then just a weariness overall, I'd say. It's not every day, but it happens here and there. |

8

9

10 Supplemental 4. PROTECTIVE THOUGHTS

| **Theme** | **Definition** | **Example** |
| --- | --- | --- |
| Inspired by and/or Proud of Colleagues | Feeling inspired, proud of, or impressed by creativity, ingenuity, and/or resilience of their colleagues | PGY3: The staff were all stretched thin. I think that was definitely a time where we kind of were all on the same goal, and so we all worked really hard. I had already built pretty strong relationships with the nursing staff on both sides of the street, and fortunately that just followed through when I was working there. If anything, I was just really concerned about so many nurses who were of a high risk age who were still working as well as the attendings. So I would just be very concerned about them getting sick. But yeah, I mean, I kept saying the nurses were the ones that really should be praised because they were the ones who were literally next to the patients all the time, exposing themselves the most out of anybody. And I really appreciated that and I always tried to highlight that. |
| Desire to or Relief of Getting Infected | Feeling relief of getting COVID, or wanting to get COVID, including when someone mentions wanting to get sick to “get it over with” or | PGY1: I honestly wanted to. I want it to get over with, I want it, I want it to get over with. I saw SARS CoV-1 before and I was not sick. I |

|  | feeling relief after they became sick so that they would not have to worry about PPE so much anymore given presumed immunity | hope if I get sick with this one, it's going to be fast and smooth. I heard [another resident’s] story, because he got sick before me. I knew that whatever we do or whatever PPE we put, we’re exposed, we're next to each other. We're not in a Covid-free bubble and whatever we do, whatever measurements were taken there, there's a big chance of me smelling it, eating it, touching it, but I really wanted to get it over with. One of the happiest days of my intern year was the day that I felt that: okay, I got it over with. |
| --- | --- | --- |
| Identifying Sources of Emotional Support | Feeling supported emotionally, feeling comfortable asking for help at home and at work, including feeling supported by friends, families, a therapist or other medical professional, other staff, or through wellness activities | PGY1: We were all experiencing that same thing, a lot of the attendings, having had past practice for 20 plus years, had never experienced anything like this in their life. You know, so what I was experiencing is the same thing they were experiencing and we were able to have that in common and everyone came together, and we worked through it, you know, I've had admin, checkup on me, you know, I've had my attendings check up on me and it just like, it gave me a sense of a family more than I had even experienced before.  We had some psychiatrists that came to the ER. They were mostly there for the patients, but they were also open to listening to us if we had any issues. I started to talk to her and I went to the corner because it started to get a little bit more personal. And I was literally able to stand there for 10, 15 minutes and vent to her about the things that I was experiencing and how I was feeling. You know, and she validated everything that I was feeling. Having them physically in the ER was extremely helpful. |
| Appreciative and/or Humbled | Feeling appreciative or humbled in a positive or optimistic way, including finding a “silver lining” in an otherwise challenging experience | PGY2: You don't expect your admin people to be there on the ground every day. So it was very excellent to be able to see that they were there to know what was going on and to actively, in real time, try to find solutions. And so I felt that, generally on our side, they were there to try to make the situation better and better for the patients. But they're limited. We don't have the money, we don't have the resources and there's only so much they could do. But I think that they did a good job in the sense of just trying to deal with the situation and given what the resources were. Same thing with our program, us program directors, they always had our backs, always made us feel like someone was out there to protect us and look out for us. We never felt that they were silent or ignoring us. . . So, I feel like we were lucky to be in the program that we were...that we had that much support from our leadership. |
| Finding Learning Opportunities | Feeling a positive emotion when speaking about finding learning opportunities in an otherwise challenging time period | PGY1: I'm not well versed in palliative care. I did a rotation in med school for that but it was a brief one. I think I was realizing the necessity for having those goals of care discussions with patients, with family members: what did they want, what do they enjoy, a brief history of what their life has been before the pandemic and what they were looking forward to and having those kinds of "code [status]" |

|  |  | discussions: would you want us to do CPR on you, would you want us to intubate you and put you on machine, and what kind of aggressive measures would you want us to take for you... and also explain that as well as to family members over the phone. I think that was a learning experience...being able to document that clearly for those patients, so you can advocate for those goals of care discussions when the time comes. |
| --- | --- | --- |
| Camaraderie and/or Teamwork | Feeling a positive emotion when speaking about the camaraderie or teamwork in their description, including description of friendship or having reciprocal thoughts and feelings of support for colleagues in the clinical environment | PGY2: I remember working well with the nurses, you know, I think there was somewhat of a shift. I remember from intern year, you know, an occasional conflict over things like drawing blood or something like that. But I don't remember that ever happening in COVID times. I remember working with them to be seamless. I actually think the whole event, as traumatizing as it was, was probably good for the camaraderie between us, at least doctors and nurses and other staff; at that point, we were sharing food deliveries and other wellness gifts that we had received from the community. I actually think we had a pretty good relationship at that time, myself and the other clinical staff members. |
| Pride in Work or Sense of Duty | Feelings of pride in work or a sense of duty to work, including feeling motivated to work hard and complete assigned and unassigned work tasks because of altruism and self- sacrifice for the good of others or to reduce the collective burden of work to be done. This carries the sentiment of "we did what we did because we care about patients and we like to save lives and make people feel better” or "I'm happy to work, I'm proud to work, and I don't mind working extra if there is extra work to be done.” | PGY4: I felt like at least on the ground level, in a day to day ED interaction, I think [the ED] developed the level of cohesion that we never had before. I think a lot of people stepped up to do something that was really hard for them to do and in a way that I'm really proud of [the ED] for doing. I think nursing and all the residents and the rest of the staff, especially on the ground level, really came through in a way that was pretty surprising. Not that I didn't love everyone before, but it was really nice to see that. So I think we ended up working well together and I think in the end, everyone, put care of patients above everything else, which in the end could have been somewhat harmful to us, but was probably the right thing to do. |
| Acquiescence or Acceptance of Reality | Accepting the reality of the situation and acknowledging or coming to terms with working during this trying time, and that the providers and team are utilizing all available resources available to them | PGY2: I feel like I'm still pretty high risk. I don't know. I feel a little radioactive compared to the rest of the world. So, you know, I don't want to see my mom. I don't want her to get it from me. Now our lives have changed. And, in the complexity of our work, we have to give up a lot of the things outside of work, the way the rest of the world did too, so we're not special. We have to give those things up. |
| Hopeful and/or Optimistic | Feeling optimistic or hopeful, about the betterment of society or workplace wellness, including creating a positive atmosphere in the workplace, and looking forward to normalcy after the pandemic resolves. | PGY4: I think down the line, as residents and also all the attendings that took care of patients during this time, we'll always remember this. And I think it'll probably affect how we practice medicine and what kind of clinicians we are for the rest of our lives. And I don't think we will really realize what effects those are until the future. But I think that there'll be a lasting impact on us. I just don't know how it's gonna impact us. Hopefully, it's for the positive. |
| Empathy for Patients and their Families | Feeling empathetic or pity towards patients | PGY2: The patients didn't have any family |

|  | and their families, including connections made during a difficult time, connecting with patients over a short period of time, and reflecting on losing patients | around them, so even if it was just holding a hand or putting a hand on a shoulder during it, during the five seconds that some of them had to gulp down a cup of water before they had to put their BiPAP mask back on, or to contact family members for them, just to give them a heads up or give them an update and then let the patient know that: alright, I spoke to your son or I spoke to your daughter- they want to let you know that they love you, and they're thinking about you and you're in their thoughts. Just little things like that. What really was the most impactful, at least from my perspective, was showing them humanity during that and trying to remember to not be so caught up in the mass craziness of it all, and truly try to take each patient for their specific situation. |
| --- | --- | --- |
| Identifying Strategies for Self Care | Identifying strategies of self-care, including religion, mindfulness practices, enjoying time off work, as well as focusing on personal wellness, health, and hygiene | PGY3: I watched a lot of Netflix, a lot of non- serious shows just to take my mind far away to something completely opposite from the heavy, depressing things we were seeing at work. So I did that a lot during the month of April. Then I got a bike during COVID also because it was just easier to get around. So then I would just bike around the city and go to the park where it's nice and peaceful and usually sunny out. And so it just feels like worlds away from the hospital and the ER, where everyone is dying. |
| Well-prepared or Confident | Feeling well-prepared or confident in decisions and abilities, including describing self-trust | PGY1: Between me and myself, I started looking at it like this is prime time. I definitely heard one of our chiefs at that time, he was comparing it to a sporting event or like an exhibition where you train for a long time and then now it's time to perform. I felt that: okay, I've been taught medicine I've been reading  all this stuff all my life. right now it's prime  time, right now is the time to be on my 100% game. |
| Supported by the Community | Feeling connected to the community and/ or feeling validated or trusted by the community and patients, including connections made through food donors, receiving thank you cards or notes, conversations with members of the community, and 7 pm cheers | PGY4: I think most people become a doctor because they want to do good. It's not always easy for sick people to show appreciation because they're consumed with their own illness and what's going on. So a lot of times I feel like we have somewhat of a thankless job. You know, sometimes we don't even get to really talk to the person or interact after the fact. And so having that level of community support every day was a clear reminder, it was a very obvious reminder that people appreciate us and what we were doing. And so it was a very positive experience in that I felt very good about myself and I felt that it just reminded me why I want to do this. Why I went into this field. |
| Feeling Useful or Helpful | Feeling useful or helpful, including finding utility or ways to be useful, being flexible with roles by working in unassigned areas, and finding time outside of work to help wellness and response efforts for the residency and hospital | PGY2: The other residents were coming up with the makeshift CPAP masks for our Bipap patients who couldn't access it. So that feeling of not being the help, but then: okay, what can we do? What can we do to find other solutions that were at least something productive that |

|  |  | we could work on while these other infrastructure issues were something that we weren't able to address? That was more something the administrators had to figure out. So some [examples] would be creating these bubble CPAP devices to kind of hold over the patients, or going across the street and asking to see if we could swap O2 tanks with the areas that had available oxygen tanks. So I guess these things sort of, even though we couldn't do anything, it made us feel like we were actively trying to find a solution. |
| --- | --- | --- |

11

12

13 Supplemental 5. CLINICAL LEARNING

| **Theme** | **Definition** | **Example** |
| --- | --- | --- |
| Self-directed Learning | Learning on one’s own as opposed to being taught by someone else | PGY3: I felt very motivated to try to read all the literature, published, journals and people blogging or sharing tweets about their insights and fighting the disease. Cause it's interesting how much information was coming out and being disseminated so quickly. Obviously, some of that turned out not to be correct, for instance, giving everyone hydroxychloroquine and azithromycin. We did that for a while and I don't think there's any evidence to support that. But it's definitely an interesting time and something I'll remember for the rest of my career.  I think whenever a paper would come out, I would read it pretty quickly, within like a day or two of it coming out, which I definitely don't normally do for medical literature. I feel very connected to it because I felt like I don't know exactly what's going on with this.  Normally, I'm not a person who reads multiple articles every month. |
| Peer-Learning | Learning from informal discussions with other residents or peers | PGY4: Regardless of what it was, we were still doing the types of training and stuff that we did: ultrasounding patients, learning from that, learning from the things that worked and the things that didn't work, learning from our colleagues who say- Hey, I realized this is a better way for us to do hypoxia rounds; this is some other stuff. And it became more like peer learning, co-learning, and I think that the thing that stuck out to me the most was at least from the senior resident perspective was when I was working with my attendings, they would be honest and say, we don't know how to manage this as much as everybody else and we're all in this together and learning, and as we would learn knowledge, we would share it together. We would figure it out. |
| Resident Oversight | Discussion of supervision of residents, and bedside teaching versus working more independently, autonomously, or with decreased supervision, including commentary on accessibility to senior residents and attending physicians | PGY1: I was taking care of very sick patients on my own in the main ED out of necessity- DKA patients, because we didn't have any more rooms since the Critical Care and Trauma area. I was literally managing them with IV pushes of insulin and my iPhone timer and barely oversight because it was so busy; it was resource driven- there is a limited |

|  |  | amount of resources. That was me pushing myself and taking on my own; it wasn't because someone [told] me to do it. |
| --- | --- | --- |
| Experiential Learning | Learning through direct patient care or involvement in clinical activities as opposed to informal discussions, supervision, didactics, or independent research | PGY4; I think that no one had seen this before. And so anything in medicine, especially in emergency medicine, for me, the things that make me nervous are the things that I've never seen before. Over the course of a career in emergency medicine, I think that you see most things. That's why you have people that have been doing this for 20 years, really don't bat an eye at a lot of different, crazy things that happen. This is no different. So initially, there's an anxiety of- “when is this going to end and how is it going to continue to escalate? What are we doing?” And now that we've sort of seen this once, I think that there's that comfort of like we got through it… makes it much easier to manage like the acute anxiety of what's coming in. And that's why I think that, at some level, at least, especially in New York, all of the physicians that dealt with COVID will operate at a level of a little bit more sanity, organized, if and when it comes back. |

14

15

16

17 Supplemental 6. WELLNESS ACTIVITIES

| **Theme** | **Definition** | **Example** |
| --- | --- | --- |
| Zoom Hangouts | Informal hangouts with other residents on virtual meeting platforms | PGY2: As part of the wellness team, some of the things that I was trying to create- one was a zoom movie night, which I don't think ended up happening, but what did come out of that was that 20- 25 people were on the *Zoom* call, just drinking and talking. It was set up for the residents by the residents. Some of us talked for like seven or eight hours on that thing because we missed each other. Different mini fellowship meetings and journal clubs and stuff like that happened over *Zoom*. We also had social hours or various check-ins that were set up by our chiefs, just to kind of check in on us and hang out and drink over *Zoom*. It was nice to still feel connected with that. |
| PPE Procurement | PPE donations to increase access to PPE and how that helps residents feel more comfortable at work | PGY1: At the very beginning, we all felt like there weren't enough. There were days when you would use the same n95 for several days, because there just wasn't enough to have one every day or every other day. So once that was settled and we were able to get more, of course that situation got a lot better. We also had the administrators get us respirators and PAPRs- after that and we felt a lot more protected than at the very beginning because nobody was prepared. I just feel like no one in the hospital was prepared for what we were about to see. But being able to act so quickly and give us the PPE that we needed, later on, was something that they did that I felt changed the situation a whole lot. |
| Days Off | Activities residents participate in on their days off, including pre-planned days off scheduled | PGY2: It was being incredibly self-indulgent- whatever kind of TV you wanted to watch: |

|  | by chiefs | something that was going to be sad and made you want to cry; something totally brainless. I didn't want to exercise. I was not going to push myself. If I didn't want to read, I didn’t push myself. For me, I was like, I'm going to take bubble baths. I took baths like four times a week, just like creating something separate from work, where you could just kind of relax and disconnect. If you wanted to eat ice  cream--I mean I ate more ice cream in the last four months than I have in my whole life. It was just kind of whatever you wanted to do. |
| --- | --- | --- |
| Food Deliveries | Free food deliveries from local businesses to residents and other healthcare team members at the hospital. | PGY3: It was nice when I didn't have to worry about food. I didn't buy groceries I came to  work and food was here. There's plenty of food. I brought back food and there was still plenty of food the next morning when I came back. It made focusing on work and focusing on the other stuff a lot easier. Let me breathe. Let me go for a run if I can. I don't need to worry about cooking, cleaning. I'll come to  work and I want to come here. It made the shift so I'm not hungry or starving. I ate better during COVID than I have any other time during residency. |
| Gift Bags or Donations | Gift bags and other items donated to residents and other healthcare team members at the hospital | PGY2: We had a whole wellness committee. We had a huge surplus of donations from various organizations to try to improve our wellness and having certain individuals distribute them and organize them. And really selflessly trying to maximize other residents’ wellness was a beautiful thing to see., Same thing with food, you know, organizing, making sure that people always have food on shifts,that's pretty cool to see. |

18

19

20 Supplemental 7. ADVICE FOR OTHER RESIDENCIES

| PGY 1 | -Pushing people to take the time that they need to, if they need it  -Providing surge residents so that there are always more people around than necessary to help with those ancillary staff tasks of replacing O2 tanks, etc  -Providing you with time off  -Providing us with the opportunities to talk about what was going on as a residency program  -The food and stuff was also really nice and it was a way to connect with the community, which was also crucial during that time period |
| --- | --- |
| PGY 2 | -The main thing with the program is that you have to protect your residents and remind them to protect themselves  -Giving them proper PPE  -Giving them time off  -Helping them figure out some kind of a work life balance or focusing on wellness and making sure they eat and stay healthy  -I think it's important that they focus beyond the residents. I think we all came into this for a reason. I think a lot of us went out of our way, and we'll continue to go out of our way for our patients and put our lives at risk for our patients. And I think the responsibility of the program is to make sure that they help protect the residents so that they can be there longer to protect these patients. |

21

| PGY 3 | -Transparency coming from the top would be really helpful, from the residency program leadership and then also the hospital and ED administration  -Fostering a supportive, connected environment where people feel empowered to voice their concerns and questions would be helpful |
| --- | --- |
| PGY 4/5 | -The biggest thing is to monitor your mental health in these situations and it's okay to not feel okay  -Not everything is going to be good if you ever have to face a situation like this, that’s okay...Just acknowledge it and if you need help, just seek help |

22

23

24

25

26

27

28

29

30

31

32

33

34

35

36

37

38

39

40

41

42

43

44

45

46

47

48

49

50

51

52

53

54

55

56

57

58

59

60

61

62

63

64

65

66

67

68

69

70

71

72

73

74

75

76

77

78

79

80

81

82

83

84

85

86

87

88

89

90

91

92

93

94

95

96

97

98

99

100

101

102

103

104

105

106

107

108

109

110

111

112

113

114

115

116

117

118

119

120

121

122

123

124

125

126

127

128

129

130

131

Supplemental 7. FINAL INTERVIEW GUIDE

1. How are you feeling? Possible probes:

a)What’s work like nowadays For graduated residents:

1. How’s the new job treating you?
2. How’s your new workplace
3. What was your experience like in the Emergency Department prior to the outbreak of COVID?
4. Thinking back to your time in the Emergency Department between the last two weeks of March and first two weeks of April, describe your experience working there.

Possible probes:

1. You mentioned feeling [burned out, overwhelmed, feeling exhausted, feeling supported, feeling well-trained].
2. Say more about that.
3. How did your experience in the Emergency Department during this period compare or contrast with your experience in the Emergency Department in prior months, if at all?
4. Describe your experience with patient care during late March and early April?
5. Describe your relationship with your patients in late March and early April, if any.
6. Without disclosing any PHI can you provide an example?
7. Describe your relationship with Emergency Department administrators and attendings in March and April, if any.
8. Do you have an example?
9. Describe your relationship with other ED staff in March and April.
10. Do you have an example?
11. Describe your training and education experience during the COVID outbreak. i)You mentioned . Say more about that.
12. Describe your relationship with your co-residents in March and April.
13. Do you have an example?
14. During this period of the last two weeks of March and first two weeks of April, what was on your mind as you arrived on shift?
15. Describe your social support during March and April. a)Say more about family support
16. Say more about peer support
17. Say more about residency support
18. Describe actions, if any, that were initiated at the workplace and in residency activities for the purpose of quality improvement or wellness during March and April.

Possible probes:

1. What was your experience with, or thoughts of them?
2. How did food deliveries affect your experience if at all?
3. How did the PPE provisions affect your experience if at all?
4. How did mental health services offered (weekly wellness sessions, therapy sessions) affect your experience if at all?
5. How did aid initiatives by the medical students’ affect your experience if at all?
6. How did virtual conference affect your experience if at all?
7. How did the [topic mentioned by the participant] affect your experience if at all?
8. Is there anything else you would like to add? It can be something mentioned prior or a topic that we did not cover or that you expected we might cover?
9. If you had to give advice to another residency program on how to manage this well for their residents, what would you say?
10. Anything else you would like to add?
